# Engineering mosquito population for vector control

**DOI:** 10.1186/1475-2875-11-S1-O44

**Published:** 2012-10-15

**Authors:** Nikolai Windbichler, Roberto Galizzi, Austin Burt, Andrea Crisanti

**Affiliations:** 1 Imperial College London Department of Life Sciences, South Kensington Campus, London, SW7 2AZ, UK; 2 Imperial College London Centre for Population Biology, Silwood Park, Ascot, SL5 7PY, UK

## Background and Results

The development of genetically engineered malaria-resistant mosquitoes has shown, as a proof-of-principle, the possibility of targeting the mosquito’s ability to serve as a disease vector [1,2]. The translation of these achievements into control measures relies on the availability of an effective gene drive technology to spread a genetic modification from laboratory mosquitoes to field populations. We have suggested that homing endonuclease genes (HEGs), a class of simple selfish genetic elements, could be exploited to develop vector control strategies aimed at spreading in a target population either novel genes that impair the mosquitoes ability to function as vector for malaria or genetic modifications that disrupt their reproductive capability [3-5]. To assess the ability of HEG based constructs to spread a genetic modification into target mosquito populations we have generated transgenic mosquitoes carrying a synthetic genetic element containing the l-*Sce*l homing endonuclease selectively activated in male during spermatogenesis. We show that the l-*Sce*l element is able to rapidly invade receptive *A. gambiae* cage populations, validating mathematical models for the transmission dynamics of HEGs. Molecular analysis confirms that the expression of l-*Sce*l in the male germline induces high rates of both cleavage of receptive chromosomes and gene conversion, which results in the gain of the l-*Sce*l gene, and underlies the observed genetic drive. Furthermore we also show that different HEGs can be engineered to reprogram their sequence specificity to selectively target *A. gambiae* sequence. These findings provide a new perspective for the implementation of genetic control measures by demonstrating a mechanism by which linked genes could be spread through vector populations. Genes that interfere with *A. gambiae* ability to transmit *Plasmodium falciparum* malaria without unbalancing key mosquito physiological processes are yet to be found, however candidates genes that impair mosquito reproductive capability are potentially available. In previous reports we showed that the homing endonuclease l-Ppol recognizing a unique site within the *Anopheles gambiae* 28S ribosomal genes could be used to selectively target X chromosome carrying spermatozoa. Our data demonstrated that in heterozygous males, the expression of l-Ppol in the testes induced a strong bias toward Y chromosome-carrying spermatozoa. However these male mosquitoes also induced complete early dominant embryo lethality in crosses with wild-type females. Irrespectively of the inheritance of the l-Ppol the spermatozoa carried a substantial amount of l-Ppol protein that attacked the maternally inherited chromosome X of the embryo. Here we show that transgenic male mosquitoes expressing a destabilized form of l-Ppol during the process of spermatogenesis generated vital male only progeny thereby decoupling the sex distortion and the embryo lethality phenotype resulting from targeting the X chromosome. Our results show how, using sequence-specific genetic drive elements like HEGs, the step from the genetic engineering of individuals to the genetic engineering of populations can be taken.
